# Flexible catheter optical coherence tomography of the porcine middle ear via the Eustachian tube using a 3D-printed reflective objective

**DOI:** 10.1117/1.JBO.30.7.076002

**Published:** 2025-07-03

**Authors:** Clayton B. Walker, Kevin Beckford, Zihan Yang, Jinyun Liu, Wihan Kim, Tomasz S. Tkaczyk, Brian E. Applegate

**Affiliations:** a University of Southern California, Caruso Department of Otolaryngology – Head and Neck Surgery, Los Angeles, California, United States; b Texas A&M University, Department of Biomedical Engineering, College Station, Texas, United States; c Rice University, Department of Electrical Engineering, Houston, Texas, United States; d Rice University, Department of Bioengineering, Houston, Texas, United States; e University of Southern California, Alfred Mann Department of Biomedical Engineering, Los Angeles, California, United States; f University of Southern California, Ming Hsieh Department of Electrical and Computer Engineering, Los Angeles, California, United States

**Keywords:** additive manufacturing, optical coherence tomography, catheter endoscope, two-photon polymerization

## Abstract

**Significance:**

Cholesteatomas, benign tumors that grow in the middle ear, can lead to conductive hearing loss. If not completely removed during surgery, these tumors may regrow. Current imaging technologies struggle to detect residual tumors noninvasively due to limitations in contrast and resolution, often necessitating additional surgery for inspection. To address this, we developed a catheter endoscope capable of being inserted through the Eustachian tube, allowing detailed examination of the middle ear without surgery. Using two-photon polymerization (2PP) technology, we fabricated miniature, side-viewing reflective endoscope objectives. This approach enabled the rapid production of single-element objectives with highly repeatable optical properties, easily adaptable to specific imaging needs.

**Aim:**

We aim to design, fabricate, and demonstrate a catheter endoscope for optical coherence tomography (OCT) endoscopy of the middle ear via the Eustachian tube.

**Approach:**

Side-viewing, reflective lenses were designed in OpticStudio and 3D printed using 2PP followed by sputter coating with gold. Standard metrology techniques were used to verify and optimize the objective’s shape. The optical performance of the catheter endoscopes was measured with a beam profiler. Finally, OCT imaging of the middle ear of a pig via the Eustachian tube was completed using the fully assembled catheter endoscope.

**Results:**

Metrology showed the printed lenses conformed closely to the design. The catheter endoscope’s FWHM spot size had a mean ± standard deviation of 25.3±1.8  μm with a measured working distance of 1.960±0.057  mm. Volumetric OCT images of the middle ear, inner ear, and Eustachian tube were captured in a postmortem pig head using the catheter endoscope.

**Conclusions:**

The 2PP approach is fast and highly repeatable for miniature reflective objective fabrication. OCT catheter endoscopy via the Eustachian tube enabled imaging of the middle ear, Eustachian tube, and surprisingly part of the inner ear.

## 1 Introduction

Cholesteatomas are benign growths that can develop in the middle ear, potentially causing conductive hearing loss if left untreated. Complete surgical removal is essential as any residual tumor can lead to regrowth. As a result, patients frequently undergo additional surgery months later to allow the surgeon to inspect the middle ear for residual tumors. Ideally, small residual tumors could be evaluated noninvasively, eliminating the need for additional surgeries and the associated costs and risks. However, current noninvasive imaging techniques, such as magnetic resonance imaging and computed tomography (CT), often struggle to detect residual tumors due to limitations in contrast and resolution. On the other hand, optical coherence tomography (OCT) has good contrast for cholesteatoma^1^ and sufficient resolution to identify tumors in the range of 10 s of microns in diameter. The challenge in using OCT for this purpose is gaining access to the middle ear space such that the entire middle ear can be imaged. Imaging through the ear canal with an OCT otoscope^2^ only provides partial coverage of the middle ear space. To address this, we have developed a side-viewing catheter endoscope that is capable of being inserted through the Eustachian tube for minimally invasive OCT imaging of the middle ear.

Catheter-based optical coherence tomography has been used to provide high-resolution, sub-surface volumetric images of many areas inside the body that are essentially inaccessible for rigid endoscopes. In particular, side-viewing fiber-optic catheters with miniature distal focusing optics have been used to image coronary arteries,^3^ sections of the gastrointestinal tract,^4^^,^^5^ the bronchi,^6^ and more. These catheters typically use rotation as the primary scanning mechanism with some form of linear motion to create spiral-pullback volumetric images. Some of these catheters are fabricated with a distal GRIN lens to focus the beam and either a prism glued to the GRIN lens with the entire catheter rotating from a proximal rotary joint^7^ or a prism that is rotated with a distal micro-motor.^8^ Others use angle-polished ellipsoid ball lenses that attempt to correct the astigmatism introduced to the beam by the outer catheter tubing.^9^ Others have introduced the two-photon polymerization (2PP) technique as a new method for producing distal optics by printing a refractive freeform lens prism directly on the fiber with astigmatism correction included in their design.^10^ 2PP works by direct laser writing with ultra-short pulses of light to polymerize photoresin. This nonlinear process relies on two-photon absorption, in which the probability of reaching the required energy level is low outside the focal volume. This initiates a chemical reaction, polymerizing the photoresin in sub-PSF regions at the focus of the objective.

Although these methods all produce high-quality catheters, they require meticulous fabrication processes that either leave room for variability in their optical performance or require a lot of time to produce a single catheter. For applications where the spectral bandwidth is quite large, chromatic aberration or the transmission spectrum may become a limiting factor for refractive focusing optics. The 2PP technique successfully addresses many challenges by allowing for the rapid fabrication of precise, repeatable, and high-resolution structures. A reflective lens design further addresses issues related to spectral bandwidth by relying on reflection rather than refraction for beam convergence. 2PP is straightforward, requiring only a 3D model from computer-aided design (CAD) software,^11^ and offers the flexibility to iteratively fine-tune the printed optics until the desired outcome is achieved.

Recognizing the advantages of 2PP over other catheter fabrication methods, we developed an OCT catheter endoscope featuring a 3D-printed reflective lens fabricated using the 2PP technique. A reflective probe was selected due to its immunity to chromatic aberration and its ability to achieve longer working distances with the same element size, eliminating the need for a GRIN lens. In addition, the reflective lens provides precise control of the desired beam output angle without additional optics. As these probes could not be printed directly to the fiber because of the subsequent sputter-coating step, the groups of these probes were printed onto a microscope slide to be used in the catheter fabrication step.

## 2 Methods

### 2.1 Reflective Lens Design and Optimization

We had several design goals in mind for the reflective lens in this application for middle ear imaging. A full-width at half-maximum (FWHM) spot size of ∼25  μm would provide high-resolution images of the middle ear space. The reflective lens’s working distance was set to 2 mm from the axis of rotation, aiming for this distance, combined with an ∼1  mm Rayleigh range, to produce focused images spanning from the catheter’s outer surface to ∼3  mm from its axis of rotation. The last of the design parameters to be considered was the diameter of the reflective lens housing. It was important to keep the catheter as narrow as possible to ensure easy insertion through the isthmus of the Eustachian tube to access the middle ear space. In humans, the Eustachian tube is narrowest at the isthmus with a minimum dimension of ∼1.5  mm.^12^ A reflective lens diameter of 300  μm was selected to fit within a 400-μm cylinder, ensuring robust protection from the surrounding 450  μm stainless-steel torque coil that encased the optical fiber to which the reflective lens “probe” was attached. This size optimization minimized print time while maintaining a sufficient lens diameter to accommodate the anticipated beam size incident on the mirror.

**Fig. 1:**
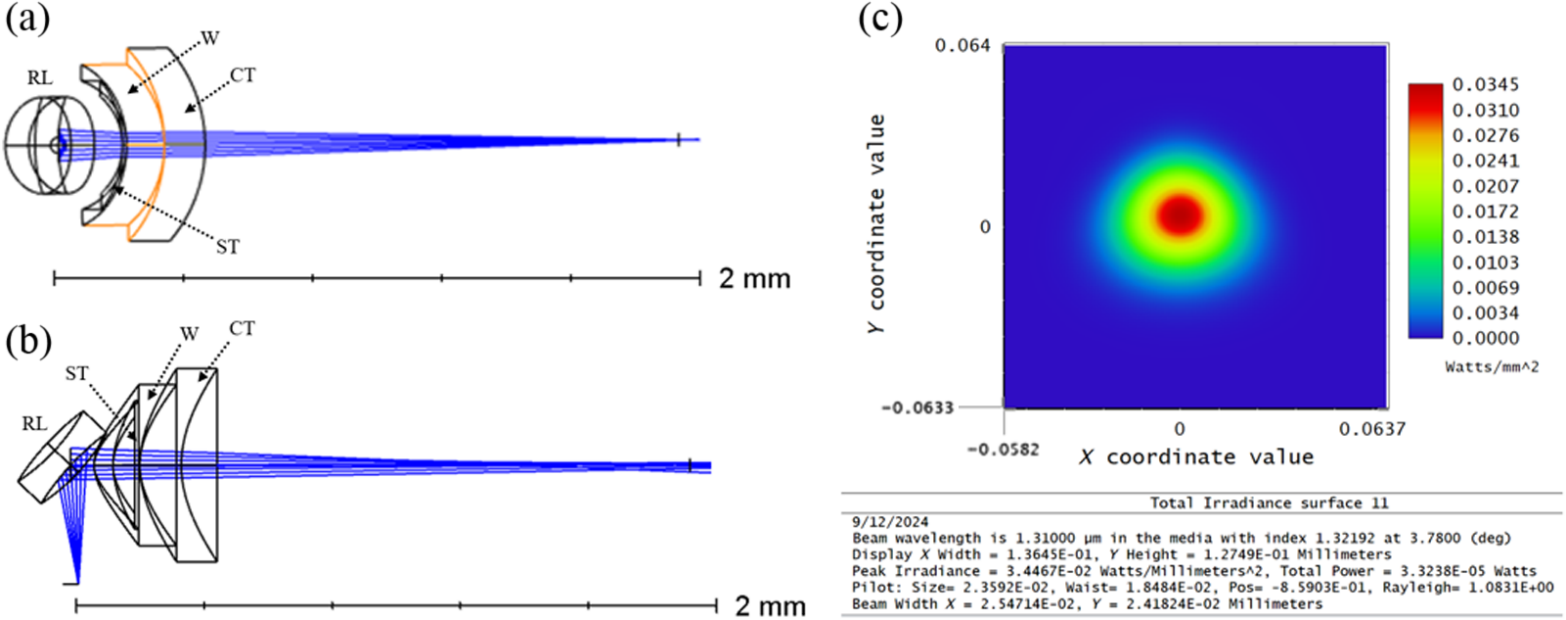
Panels (a) and (b) are orthogonal 3D views, y to z and x to y, respectively, of the optical model in Zemax. All of the components of the catheter are modeled as optical elements. Slightly different outer diameters were used for the elements to make them more visible in the drawing. Panel (a) shows the y to z view of the optical design where the output from the fiber is coming out of the page, RL indicates the reflective lens, ST indicates the shrink tubing that seals the air gap between the fiber output and the reflective lens, W indicates the water inside the catheter which is also highlighted for clarity, and CT indicates the catheter tubing. Panel (b) provides an x to y view showing a better angle of the mirror surface where all surfaces are labeled like panel (a). Panel (c) shows the enface view of the slightly elliptical beam in the POP window. It also indicates a Rayleigh range of 1.08 mm, a beam FWHMx of 25.5  μm, and a FWHMy of 24.2  μm below the plot.

The optical design, shown in Figs. 1(a) and 1(b), and optimization were done in OpticStudio (Ansys Zemax) where the reflective lens was modeled as a biconic mirror placed near the output of SMF-28e optical fiber and oriented to provide a 94-deg reflection from the central axis of the optical fiber. To make the simulations as accurate as possible, the mirror was also placed within the catheter and airgap shrink tubing—discussed in more detail in the catheter fabrication section. To account for the cleaved fiber surface being polished at an 8-deg angle—minimizing back reflections into the system from that interface—we incorporated the beam’s refraction angle from the fiber’s angled surface into the input beam’s incident angle on the mirror. There were three main variables considered in the design. The numerical aperture (NA) of the SMF-28e fiber at the system wavelength of 1310 nm was 0.13, so the distance between the mirror and the fiber output was used to control the beam size on the mirror. The radius of curvature of the biconic mirror along the x- and y-axes was independently adjusted to control the beam shape output as well as the general convergence of the beam.

We tried to optimize for both spot size and depth of field by considering the NA of the catheter endoscope toward the object plane—where the tissue would be. Constraining the Rayleigh range to be something between 1 and 1.5 mm provided the longest possible depth of field (DOF) while also ensuring the NA would be suitable to achieve the preferred lateral resolution. The last thing to consider was the correction of the beam shape because the shape of the catheter sheath acts as a negative cylindrical lens. To optimize this design, the parameters that were discussed were applied in the merit function editor in OpticStudio (Ansys Zemax, Kirkland, Washington, United States). In this case, the working f/# (WF/#) was optimized to achieve a spot size of 25  μm FWHM spec. In Fig. 1(c), physical optics propagation (POP) analysis showed a Rayleigh range of 1.08 mm, and the beam intensity FWHM along the x- and y-axes was 25.5 and 24.2  μm, respectively.

### 2.2 Reflective Lens Fabrication and Characterization

**Fig. 2:**
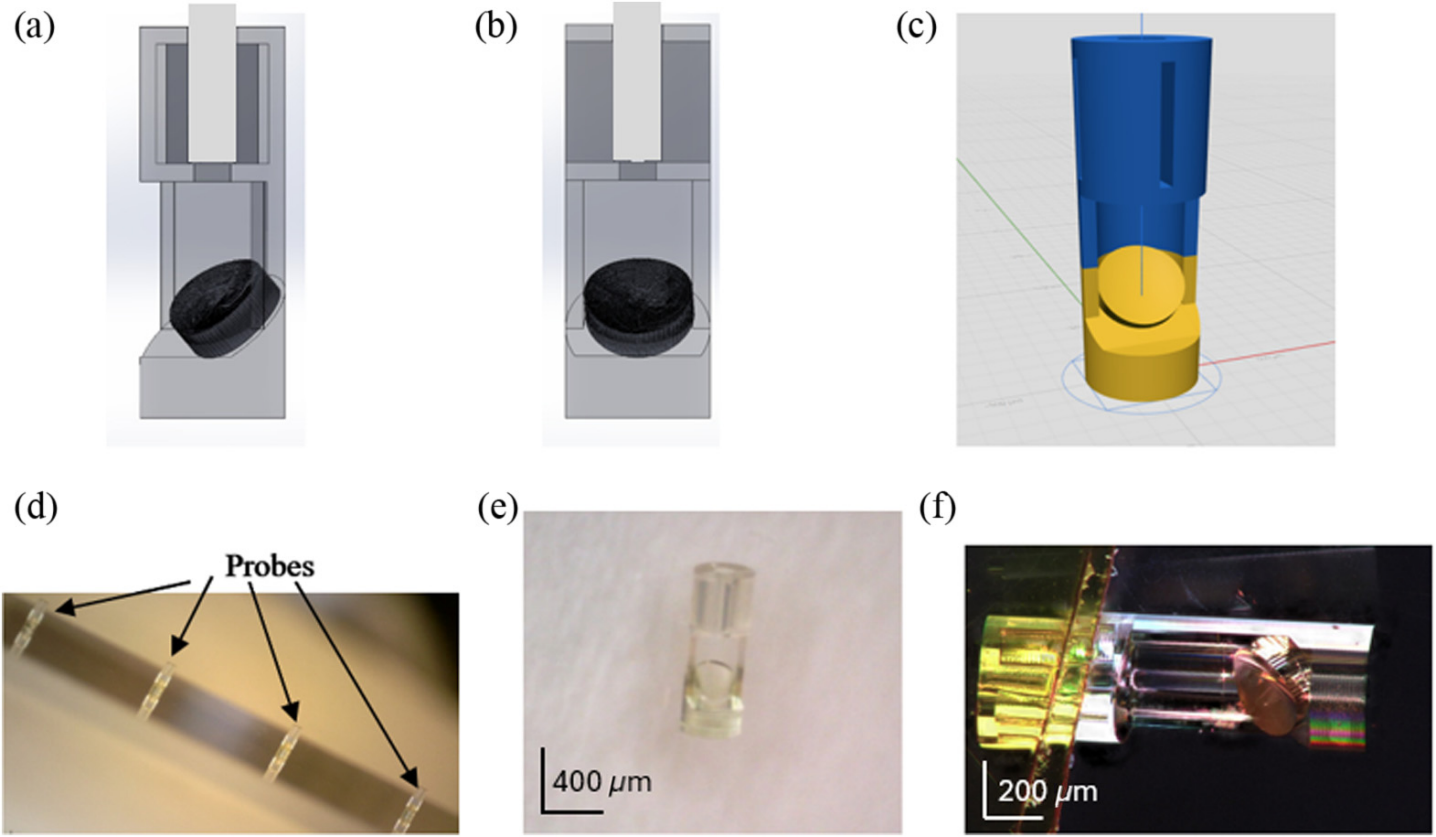
Panel (a) shows the side view of the probe. Panel (b) shows the front view of the probe. Panel (c) shows the STL of the probe in NanoprintX software. Panel (d) shows the probe fabricated in an array (e) closer look at the fabricated probe. Panel (f) is a high-resolution photo of the probe after coating.

After achieving satisfactory results from the OpticStudio simulation and optimization, the stereolithography (STL) file of the mirror was exported to add the optomechanics that would fix the mirror at the correct distance from the cleaved fiber. The optomechanics were designed in SOLIDWORKS to create a robust assembly that protects the mirror and allows for easy attachment to bare fiber. As illustrated in Fig. 2, the design features a cylindrical structure with an outer diameter of 400  μm and a total length of 1.14 mm. A cutout was incorporated to securely hold the mirror in place while ensuring an unobstructed imaging window. Once clear of the imaging window, the optomechanics rejoined to complete a hollow cylinder down to the flat, proximal end of the probe. The opening on the proximal end of the probe was 140  μm in diameter, providing a 15  μm tolerance for insertion of the fiber with an outer diameter of 125  μm. With a fiber length of 400  μm, the maximum angular deviation was 2.15 deg, resulting in the beam striking the mirror off-center by up to 13.9  μm in the worst-case scenario. A fiber stop was added at the distal end of the fiber insertion hole to precisely position the mirror 370  μm from the cleaved fiber end. Four slots—each 20  μm wide and 350  μm long—were spaced 90 deg apart around the fiber insertion channel to allow proper drainage of any uncured epoxy. As a final detail, the mirror was shifted to ensure that the beam would hit its center with the same angle of incidence as used in the simulations.

**Fig. 3:**
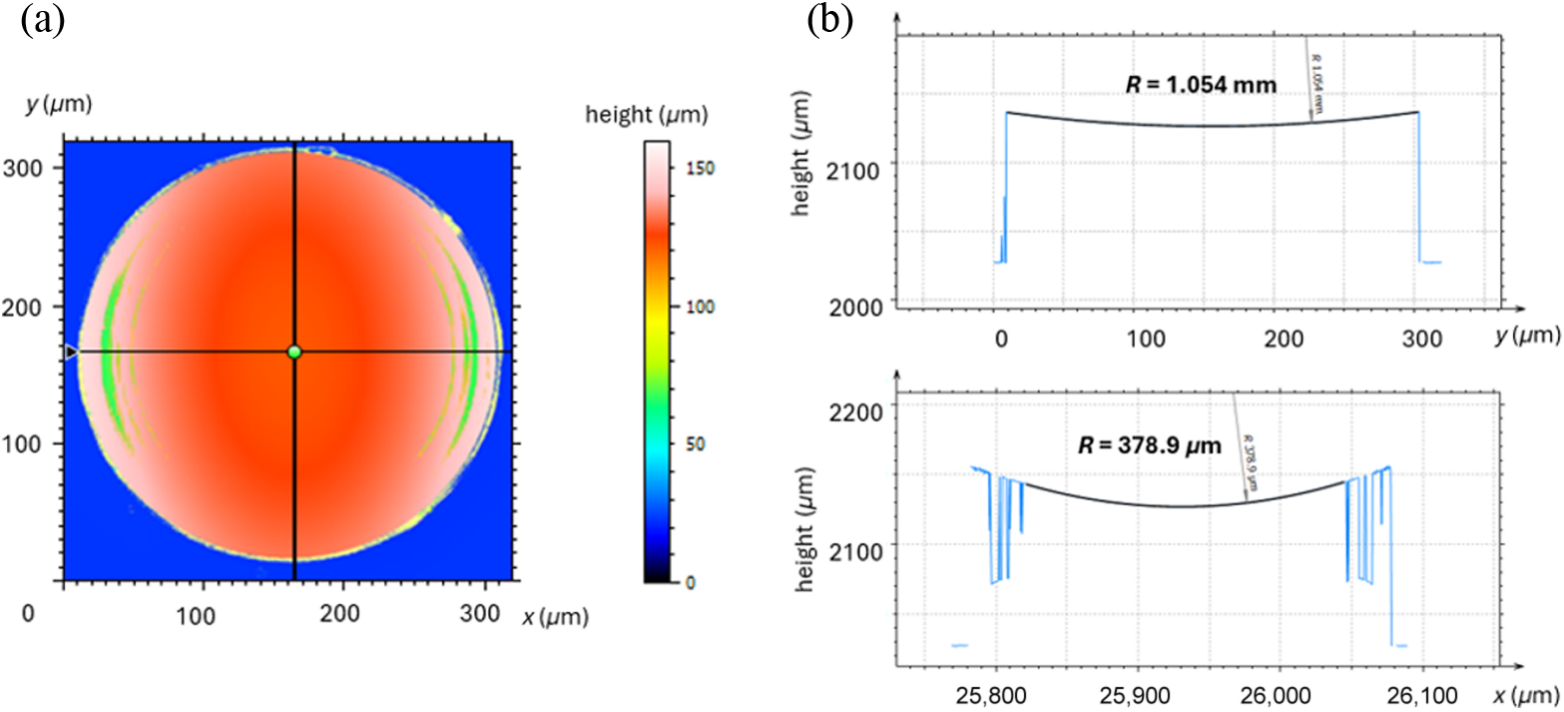
Results from a confocal metrology system used to measure the biconic surface of the reflective lens. Panel (a) shows the heat map of the topography of the reflective lens. Panel (b) shows the cross-sectional profile taken along the y-axis (top) and x-axis (bottom) through the center of the reflective lens. From these profiles, the radius of curvature along each axis was taken to compare with those expected in the design.

With a final design of the probe modeled in SOLIDWORKS, the entire assembly was saved as an STL file for 3D printing with the Quantum X (Nanoscribe, Eggenstein-Leopoldshafen, Germany). The Quantum X employs the 2PP additive manufacturing technique, with direct laser writing using ultrashort pulses of 780 nm light to cure the photoresin based on the two-photon absorption effect. To print the structure, the STL design file was converted to the Quantum X/Nanoscribe native NanoprintX format, which was then used to generate the system-specific G-code printing file, as shown in Fig. 2(c). This software allowed for the fabrication of probes using grayscale lithography (2GL). The initial attempts to fabricate the probe used DescribeX, which necessitated printing at a high resolution to get the required smoothness at the cost of time. DescribeX uses a 2PP mode where the regular slicing of layers is composed of voxels of a fixed height that are stacked upon each other to match the surface shape. By contrast, 2GL speeds up the fabrication process by tuning the laser power to modulate the voxel height so that it can match the structure shape while maintaining high precision and smoothness. This enabled a much more precise structure at a higher slicing distance, which reduced the printing time. In addition, the resolution of different parts of the structure could be optimized to shorten the printing process even more. Figure 2(c) illustrates two distinct probe sections: the bottom half, which contains the yellow lens and employs the 2GL parameters, and the top half, which uses the 2PP lower-resolution parameters without incorporating 2GL. This allowed for the fabrication of these probes at much faster speeds without sacrificing quality. For the bottom half of the print, the 25×, 0.8 NA objective was used with a 200,000  μm/s scanning speed and 70 mW laser power. A slicing distance of 1.2  μm and a hatching distance of 0.25  μm were chosen. The bottom half was fabricated with the 2GL mode. The top half used a preset of 250,000  μm/s scanning speed and 100 mW laser power. The slicing distance and hatching distance for this half were 2.4 and 0.5  μm, respectively. This top half was printed with the regular 2PP mode. With all the print settings configured, IP-S resin (Nanoscribe) was deposited onto a fused silica microscope slide that was then mounted to the Quantum X. It took 35 min to print each probe using the 2GL method, which was quite an improvement compared with the previous print using DescribeX, where each probe took 6 h to print. This allowed for an array of seven probes to be printed in one step due to the short printing time, as shown in Fig. 2(d). Post-processing consisted of developing and washing out the nonpolymerized resin with SU-8 developer and Isopropyl-Alcohol (IPA). The structure was placed in SU-8 developer for 20 min and then in IPA for 2 min. A closer view of the probe is pictured in Figs. 2(d) and 2(e) after the post-processing step. To complete the reflective lens, a 10 nm layer of gold was sputter-coated on the lenses with the Denton Desk V sputter system. Figure 2(f) shows the completed reflective lens before being packaged and shipped to USC for final assembly and testing. The mirror was characterized by a confocal profiler (MarSurf CM explorer). The radius of curvature in both the x- and y-directions and surface roughness were evaluated using a confocal metrology microscope (MarVision, Mahr) to compare with the original design values. The topography map shown in Fig. 3(a) was evaluated to find the center of the reflective lens that would act as the origin for cross-sectional surface profiles along the x- and y-axes. The radius of curvature of the mirror surface in each profile, shown in Fig. 3(b), was measured to give 1.054 mm in the y-direction and 378.9  μm in the x-direction. Both of these measurements closely resembled the design values of 1.064 mm and 369  μm in the y and x-directions, respectively.

### 2.3 Catheter Endoscope and Imaging Setup

**Fig. 4:**
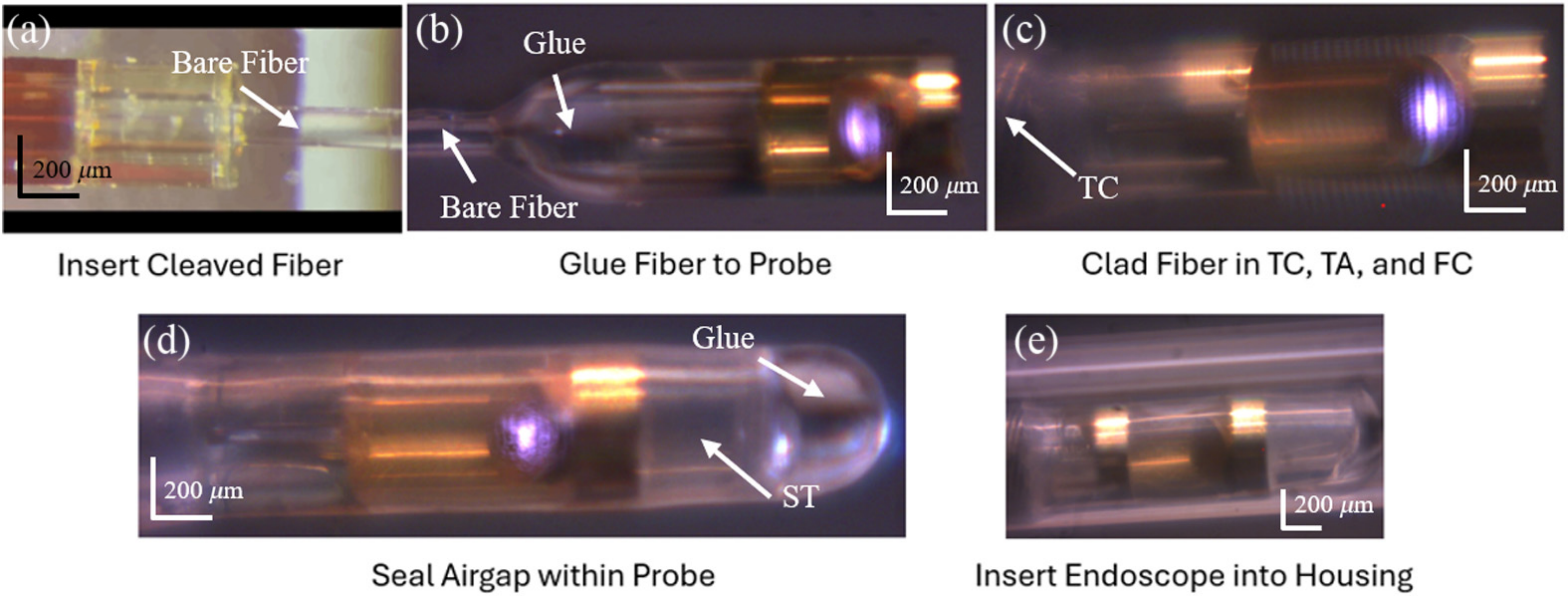
Key steps in catheter fabrication in order: (a) cleaved fiber inserted into probe, (b) glue the fiber and probe together, (c) add torque coil (TC), torque adapter (TA), fiber connector (FC), and protective steel tubing, (d) seal the airgap between the cleaved fiber and probe mirror with FEP shrink tubing (ST), and (e) insert finished catheter endoscope into catheter.

A fiber rotation mount (HFR007, Thorlabs, Newton, New Jersey, United States) was attached to a three-axis stage to hold the bare, 8-deg polished, optical fiber and match the beam angle incident on the mirror with the angle used in the simulation. Once the fiber was properly oriented and aligned with the fiber seating opening of the probe, we were able to quickly insert the optical fiber into the probe and glue it in place using UV-cured epoxy (NOA61, Norland Products, Jamesburg, New Jersey, United States) under a standard stereo microscope (SteREO Discovery, Zeiss, Oberkochen, Germany). The fiber with the probe was clad with a stainless-steel torque coil (Asahi Intecc Co, LD, Aichi, Japan) using two-part medical epoxy (M-31CL, Loctite, Rocky Hill, Connecticut, United States). After letting the medical epoxy cure, the fiber connector, torque adapter, and protective steel tubing were all bound to the proximal end of the catheter endoscope simultaneously using high-temperature fiber connector epoxy (Eccobond F 123, Loctite) that was cured at 100°C over a 15-min period. As the final step to complete the catheter, fluorinated ethylene propylene (FEP) heat-shrink tubing was secured to the optical fiber at the proximal end of the probe with UV-cured epoxy before heat and tension were applied to shrink the rest of the tubing around the probe. This provided a water-tight seal around the air gap between the mirror of the probe and the angle-polished end of the optical fiber. Figure 4 shows the catheter and probe in the sequential stages of the catheter endoscope assembly. The final outer diameter of the catheter endoscope (excluding the catheter) was ∼480  μm.

**Fig. 5:**
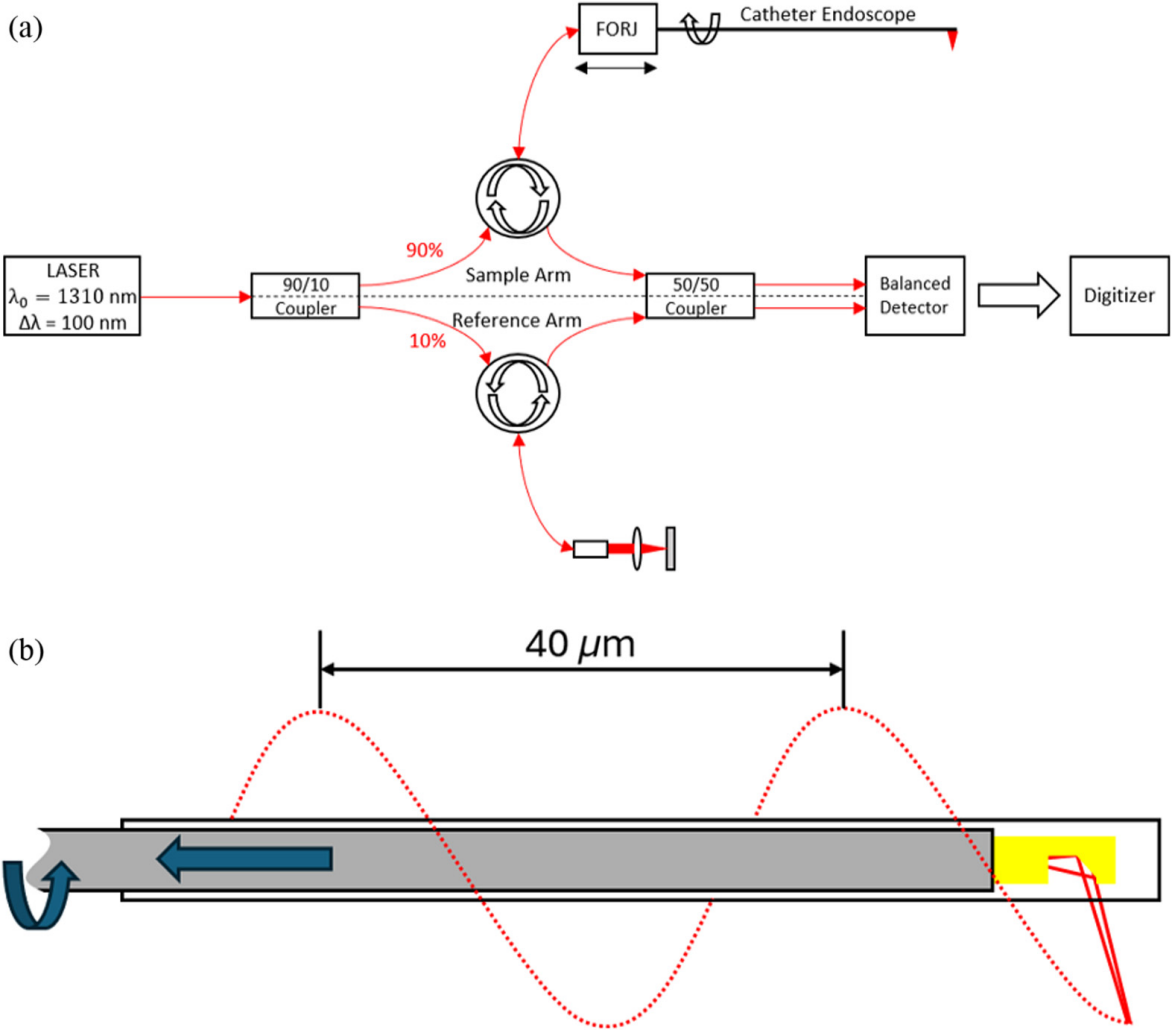
(a) Diagram of the OCT system used for imaging experiments. Panel (b) shows a diagram of the spiral pullback volumetric imaging technique. Here, the solid red lines represent the beam exiting the catheter endoscope, and the dotted red line shows an exaggerated path the beam would be scanned as it is pulled along the length of the housing and rotated. The pullback speed was chosen to provide a 12.5  μm spacing between each full rotation.

The catheter was made from a 0.5-m length of FEP tubing that served as an imaging window bonded to a 1-m length of Pebax tubing that was attached proximally to a hemostasis valve. The catheter was filled with water before insertion of the catheter endoscope. The water filled the empty space between the catheter endoscope and the inside wall of the catheter and served as a lubricant between the rotating catheter endoscope and the stationary catheter to reduce the nonuniform rotation distortion (NURD) typically found in catheter-based OCT systems. The water (n=1.33) also helped minimize any back-reflections of the sample beam by matching the refractive index of the FEP (n=1.34). The imaging window of the catheter had an outer diameter of 0.915 mm and an inside diameter of 0.66 mm, which allowed a little room for the catheter endoscope to move about inside the catheter. The end of the catheter was sealed with UV-cured epoxy to prevent water from leaking out. The catheter endoscope was then connected to our custom-built Mach–Zehnder interferometer, shown in Fig. 5(a), that included a commercial fiber-optic rotary joint (FORJ, Princetel, Inc., Hamilton Township, New Jersey, United States) in the sample arm to rotate the catheter endoscope. The system used a 100 kHz swept laser source (Insight Photonic Solutions, Inc., Lafayette, Colorado, United States) with a bandwidth of 91 nm centered at 1310 nm, providing a 14.5  μm axial resolution (in tissue, n=1.3). The signal from the balanced photodiode (BPD) was digitized (ATS9373, Alazar Tech, Inc., Pointe-Claire, Quebec, Canada) and processed using custom software written in Python, C++, and CUDA with a GeForce GTX-1080 (Nvidia). To facilitate spiral-pullback volumetric imaging, the FORJ was rotated with a DC motor (Maxon) while the catheter endoscope was pulled longitudinally in the proximal direction inside the stationary catheter sheath using an electromechanical linear stage (Zaber Technologies, Inc. Vancouver, Canada). A diagram of the spiral pullback imaging technique is shown for reference in Fig. 5(b).

In the imaging experiments that followed, the catheter endoscope was rotated at a constant 100 revolutions per second, providing 1000 circumferential samples per rotation. The pullback speed was set to 1.25  mm/s, which gave us a longitudinal sampling of 12.5  μm.

### 2.4 Performance Experiment

Once the catheter endoscope was completed, the optical performance was verified experimentally using a custom-built beam profiler. The probe’s performance was assessed by measuring two key parameters: the working distance and the FWHM spot size. Small movements of the catheter endoscope would cause it to rotate slightly, so we elected to move the beam profiler while keeping the catheter stationary. To achieve this, a 1.3-megapixel CMOS camera (uEye, IDS) with 3.6  μm square pixels and an objective with a 2.1 magnification was mounted onto a set of stages that provided 3 degrees of freedom, allowing alignment of the camera along the beam propagation direction and capture the beam profile.

The stage that was aligned along the beam propagation direction from the catheter was used to make the working distance measurements by first setting its value to 0 mm at the position where the probe mirror was in focus. The stage was then slowly adjusted with the beam monitored in real-time until the spot size appeared to transition from converging to diverging—indicating the beam had reached its smallest size, i.e., the beam waist. A grayscale image was captured of the focused beam once the integration time was adjusted to maximize the dynamic range while avoiding saturation.

The difference between the stage position for the 0 mm mark and the stage position at the beam waist was taken as the working distance of the probe. To ensure maximum precision in measuring the FWHM, the two half-max pixel positions on either side of the beam profile’s peak intensity were identified. This was achieved through linear interpolation between each pair of adjacent pixels with intensities that bracketed the half-max intensity value. The difference between these pixel positions was taken and converted to micrometers using the pixel size and magnification of the objective lens. Finally, we aimed to verify the depth of field (DOF) for each probe. To achieve this, the beam waist measurements taken at the $1/e^{2}$ intensity value of the beam profile on either side of the maximum intensity point were averaged. The Rayleigh range was then calculated using the standard Gaussian beam equation where λ was set to 1310 nm, the central wavelength of the swept laser. We opted to calculate the Rayleigh range rather than measure it directly, as real-time beam width measurements were not possible with this setup. Once the Rayleigh range for each probe was found, it was doubled to get the final DOF number for comparison with the simulation results.

### 2.5 Imaging Experiment

**Fig. 6:**
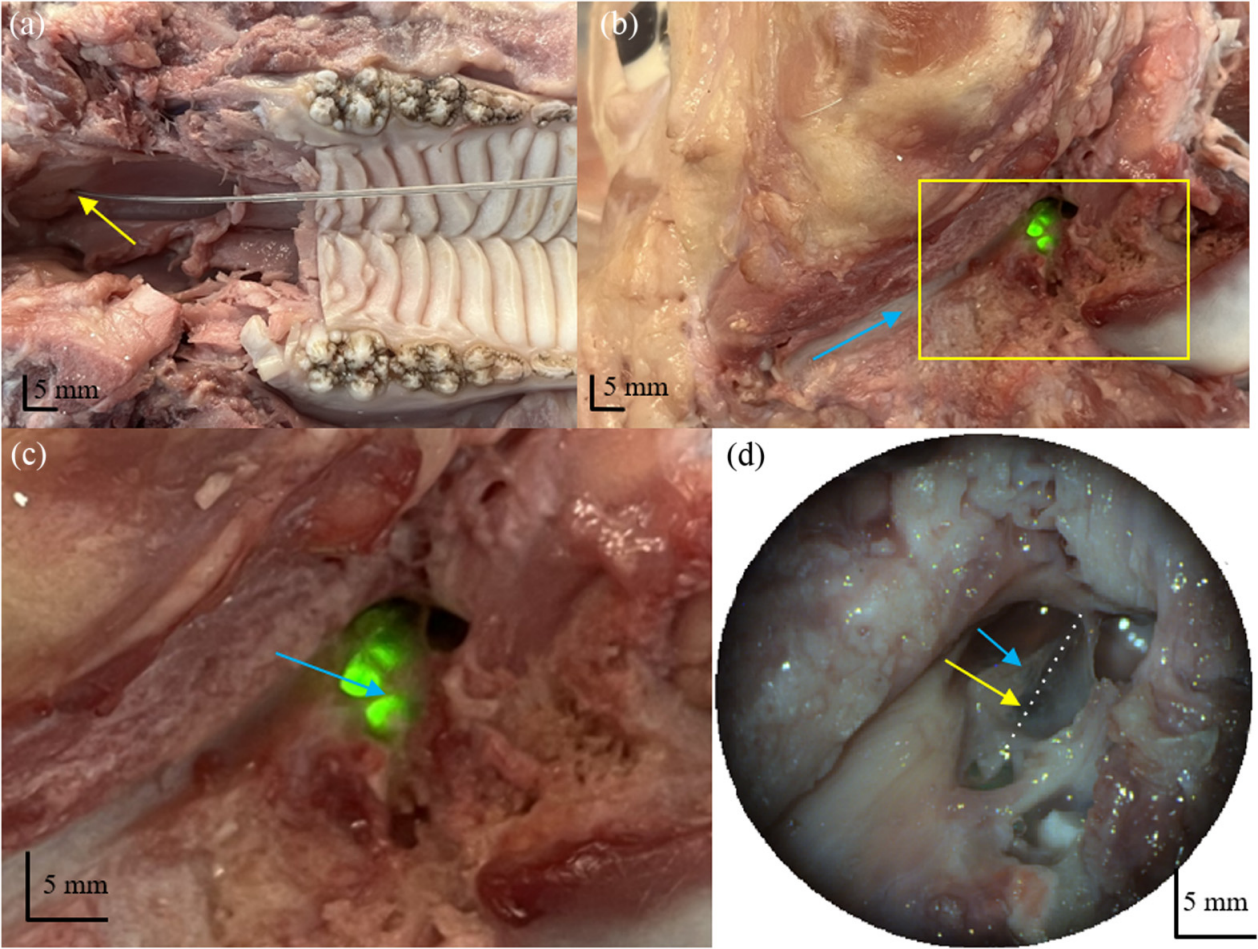
(a) Catheter endoscope being inserted through the Eustachian tube entrance as indicated by the yellow arrow. (b) Green light can be seen from the middle ear space—indicating the catheter endoscope reached the middle ear. The blue arrow points out the resected ear canal for reference. The rectangle shown in panel (b) shows the region that the magnified image in panel (c) is highlighting. In panel (c), the handle of the malleus is indicated by the light blue arrow and can be clearly seen through the tympanic membrane. (d) The catheter endoscope is indicated by the yellow arrow. The white dotted line was aligned with the visible parts of the catheter endoscope and shows that it is oriented near the handle of the malleus, which is indicated by the light blue arrow.

After verifying the optical performance of the catheter endoscope, we tested the imaging performance in a porcine cadaver head. Prior to the imaging experiment, the distal end of the catheter was left unsealed while the catheter endoscope rotated at half the intended imaging speed, and water was introduced to flush out any residual air bubbles. The distal end of the catheter was resealed once all air bubbles were removed. The intended long-term application is to image the middle ear in human patients by accessing it through the Eustachian tube. The middle ear anatomy of the pig is similar to humans;^13^ hence, the porcine cadaver head provided an opportunity to test the system in a comparable setting. The pig head was procured as a generous donation from a meat-packing facility (Glen Rose Meat Co) for research purposes. It was allowed to thaw before the mandible and lower half of the head were surgically removed to provide easy access to the Eustachian tube entrances, indicated by the yellow arrow in Fig. 6(a) in the nasopharynx. The ear canal, indicated by the light blue arrow in Fig. 6(b), was then resected back to the tympanic membrane—allowing us a clear view of the middle ear through the tympanic membrane.

This helped us to visually confirm when the catheter endoscope had been inserted far enough through the Eustachian tube to enter the middle ear space. To do this, we sent a green laser through the catheter endoscope during the insertion process through the Eustachian tube. Once the green light was clearly visible through the tympanic membrane, we positioned the catheter endoscope and collected several spiral-pullback volumetric OCT images of the middle ear space and the Eustachian tube. Figure 6 shows the insertion of the catheter through the Eustachian tube entrance in panel (a), the visual confirmation that the catheter reached the middle ear space with the green laser shining through the tympanic membrane in panels (b) and (c), and the catheter probe pushed through and into the middle ear in panel (d).

To confirm the location of the catheter endoscope and acquire a set of images for comparison with those captured using the catheter endoscope, we used our surgical microscope OCT system^14^ to image the middle ear through the tympanic membrane.

### 2.6 Image Processing

The raw volumetric data acquired by the catheter OCT system needed to be corrected for non-uniform movement of the endoscope during imaging. First, the sheath in each OCT image was identified as a reference to correct the radial distortion caused by the distance changes between the middle ear of the pig head and the catheter probe. Then, inter-frame registration including cross-correlation and nonrigid registration (similarity) was performed to correct for the potential distortion caused by mechanical friction between the catheter torque coil and the sheath or NURD. After distortion correction, a geometrical transformation was applied to the data to convert to polar coordinates.

## 3 Results and Discussion

### 3.1 Optical Performance

Using the custom beam-profiler setup, we found the FWHM of the focal spot in the longitudinal direction for each of the seven-probe set. The mean and standard deviation across the seven samples were 25.3±1.8  μm. We also found the working distance for the set of seven probes to be 1.96±0.10  mm. The Rayleigh range for six out of the seven probes was at least 1 mm with a mean ± standard deviation across all 7 of 1.11±0.150  mm. The measurements for each probe and the measurement statistics for the set of probes are given for each parameter in Table 1. The spot size and Rayleigh range were a good match with the simulated values in our OpticStudio design and were quite consistent across six of the set of seven probes.

**Table 1:** Measurements of optical performance for consistency.

	Working distance (mm)	FWHMx spot size (μm)	Rayleigh range (mm)
Probe 1	2.01	25.7	1.14
Probe 2	1.75	21.7	0.82
Probe 3	2.01	25.8	1.15
Probe 4	2.01	25.8	1.15
Probe 5	2.06	27.4	1.30
Probe 6	1.95	26.4	1.21
Probe 7	1.88	24.5	1.04
Mean and standard deviation	1.96 ± 0.10	25.3 ± 1.8	1.11 ± 0.15

To investigate whether the deviations in reflective lens performance were attributable to variations in the printed structure or if this deviation was due to catheter endoscope fabrication variability, we fabricated 10 additional reflective lenses directly onto a microscope slide in a flat orientation. We then measured both the radius of curvature in the x-direction and that in the y-direction, with the same Mahr confocal metrology system used to get the results shown in Fig. 3. The results of these measurements for each lens are shown in Table 2 below with the mean and standard deviation for each measurement being in the final row. For comparison with the measurements in Table 2, the expected values of the radius of curvature of the lenses in the x- and y-direction were 369 and 1064  μm, respectively. The mean of each measurement for the set of 10 lenses perfectly matched the expected values with a standard deviation of less than 1%.

**Table 2:** Metrology of reflective lenses for consistency.

	Radius of curvature in x (μm)	Radius of curvature in y (μm)
Lens 1	366.4	1059
Lens 2	368.4	1060
Lens 3	372.3	1074
Lens 4	366.8	1064
Lens 5	371.1	1066
Lens 6	369.7	1070
Lens 7	367.7	1066
Lens 8	370.9	1070
Lens 9	368.6	1065
Lens 10	368.0	1055
Mean and standard deviation	369 ± 1.9	1064 ± 6

### 3.2 Imaging Experiment

The *ex vivo* porcine middle ear was accessed by inserting a catheter endoscope through the Eustachian tube, allowing for the acquisition of several spiral-pullback volumetric OCT images of the middle ear and Eustachian tube. The middle ear was also imaged through the tympanic membrane from outside the ear with an OCT microscope^14^ previously developed for use in the operating room. The video image from this microscope aided in locating the catheter endoscope through the tympanic membrane with enough contextual information about the middle ear anatomy around the endoscope to help us identify structures within the volumetric image.

**Fig. 7:**
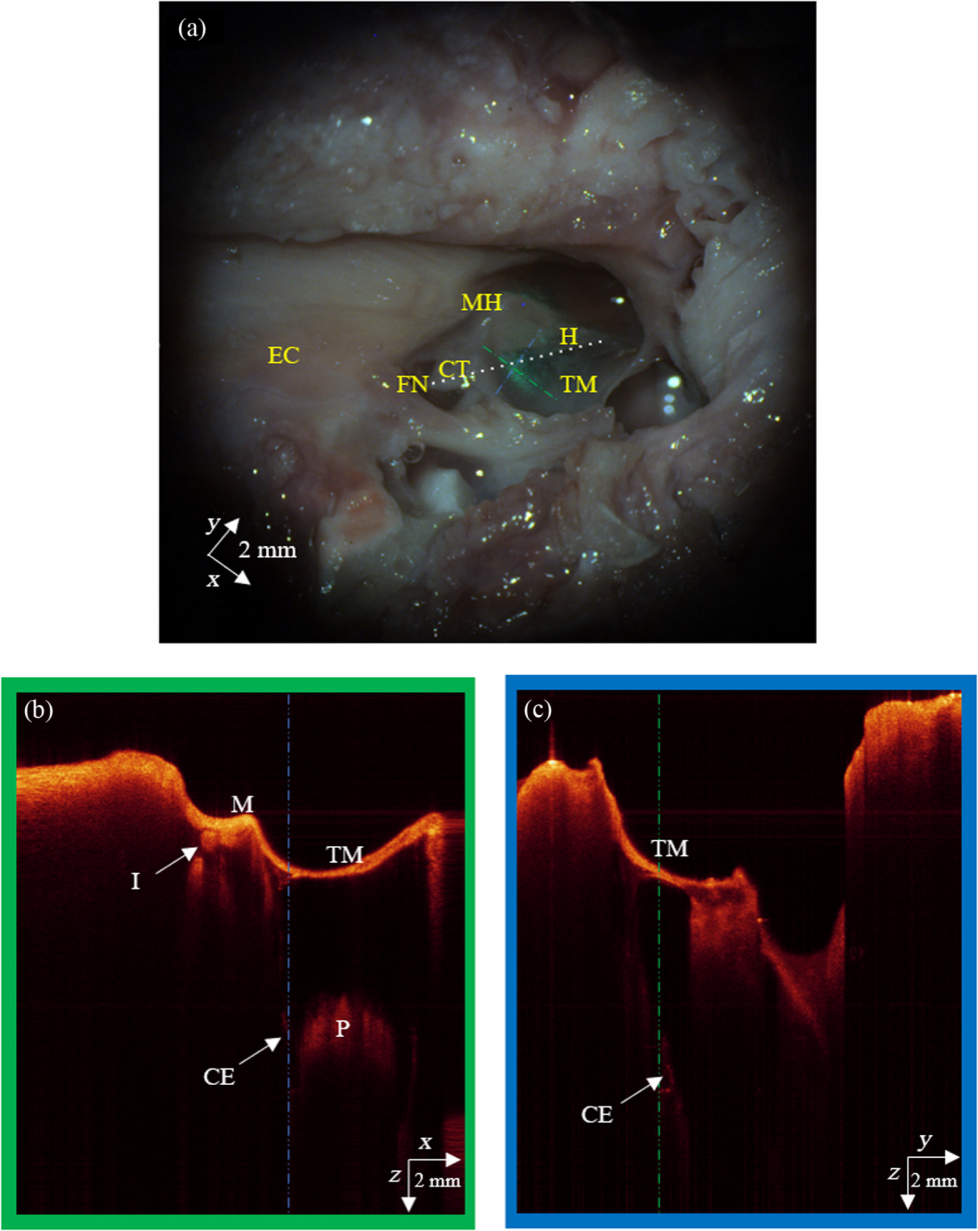
All the images here are orthogonal to each other. Panel (a) is a snapshot of the video feed from the surgical microscope that shows middle ear structures and the catheter endoscope orientation as the white dotted line. EC is the ear canal, FN is the facial nerve, CT is the chorda tympani, MH is the malleus head, H is the malleus handle, and TM is the tympanic membrane—which covers most of the right-hand side of the image. Panel (b) is the OCT B-scan taken along the green dashed line in the x to z plane where M is the malleus, I is the incus, CE is a portion of the catheter endoscope, and P is the cochlear promontory. Panel (c) is the OCT B-scan taken along the blue dashed line in the y to z plane.

Using figures from Gurr et al.^15^ as a reference, we labeled key structures within the microscope video image. Figure 7(a) presents the labeled microscope image, where the catheter endoscope, visible through the tympanic membrane, is highlighted with a white dotted line for reference.

Using the images from the OCT surgical microscope to locate the catheter endoscope, we realized that the catheter endoscope was mostly shadowed by the ossicles and structures superficial to the middle ear space. Nevertheless, we were able to capture small glimpses of the catheter endoscope to better understand its orientation between the isthmus of the Eustachian tube and the tip location near the chorda tympani. Figures 7(b) and 7(c) show orthogonal cross-sectional images of the middle ear space from the OCT surgical microscope, which both show a short section of the catheter endoscope passing very close to the cochlear promontory up toward the incudomalleolar joint along the white dotted line that labels the catheter endoscope in Fig. 7(a).

**Fig. 8:**
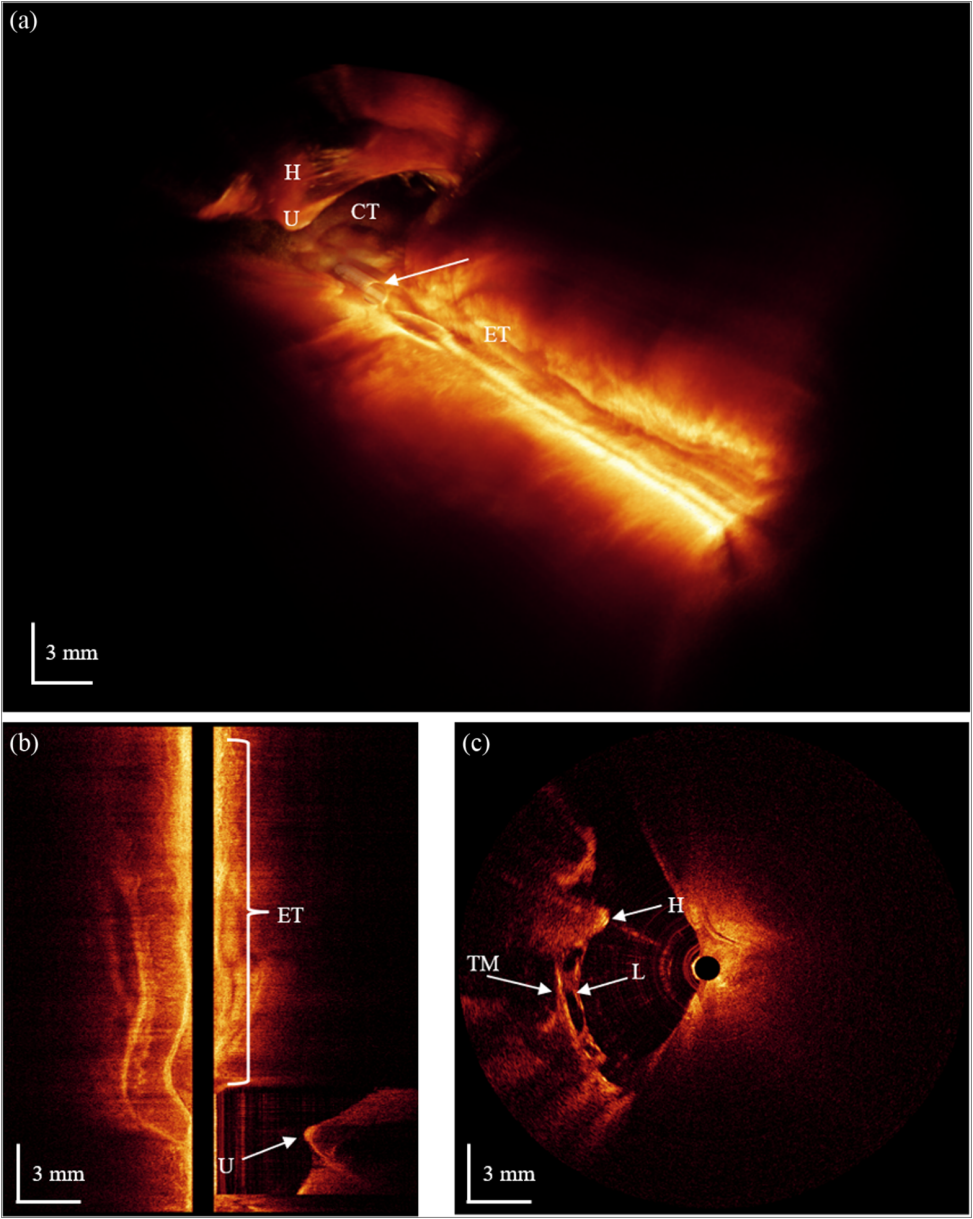
(a) Volumetric OCT image of the middle ear space taken with the catheter endoscope. Here, H is the handle of the malleus, U is the umbo of the tympanic membrane, CT is the chorda tympani, and ET is the Eustachian tube. The arrow indicates part of the catheter—seen here as a perfect cylinder. Panel (b) shows many layers of cartilage and fatty deposits along a cross-section of the Eustachian tube along with the umbo in the middle ear. Panel (c) is an orthogonal cross-section to panel (b) where the handle of the malleus (H) is seen with a lot of connective tissue (L), likely ligaments, attaching to it, the tympanic membrane (TM), and the attic wall of the middle ear.

Referring to Fig. 7(a) for guidance on the anatomical orientation of the middle ear structures relative to the catheter endoscope, within the volumetric OCT image rendered in Fig. 8(a), the handle of the malleus (H) is distinctly visible, including the umbo (U) of the tympanic membrane. The tip of the catheter endoscope is positioned near the chorda tympani (CT), in close proximity to the round window of the cochlea. With these structures in mind, we can see how the volumetric image in Fig. 8 fits with the anatomy to identify structures within the middle ear.

A strong reflection can be clearly seen at the umbo that also appears to extend furthest into the middle ear space, whereas the rest of the attached malleus handle is slightly rising out of the middle ear as it approaches the malleus head not seen in this image. An arched structure is also visible at the very distal end of the volume image, which appears to be the chorda tympani when referencing Fig. 7(a) and other literature. A right-angle cutout was used at the proximal end of this image to allow a clear view of the layers surrounding the Eustachian tube as well as to the rendered portion of the catheter.

It is important to remember that the catheter endoscope was inside the 3D image field when looking at this image as it can become confusing to understand that this is only a clear look at the bottom of structures such as the malleus handle and chorda tympani while looking at the top of the Eustachian tube entrance. This different perspective also makes it more difficult to compare Fig. 7 with Fig. 8, but using the known anatomical landmarks as a guide, Fig. 7 can be used to confirm what was expected in Fig. 8. To indicate the location of the catheter endoscope, a white arrow points to the catheter, depicted as a perfectly cylindrical surface within the volumetric image shown in Fig. 8(a). Although the primary objective was to image the middle ear space, we also imaged several millimeters into the Eustachian tube lumen. To illustrate this, Fig. 8(b) shows the umbo (U) and the thinner parts of the tympanic membrane connecting to the tympanic sulcus above and below the umbo. Figure 8(b) also shows a section of the Eustachian tube where layers of tissue beneath the surface can be seen that match the deposits of fat or cartilage, as seen in Byun et al.^16^ Imaging the Eustachian tube may be important for Eustachian tube dysfunction, which is sometimes treated using a balloon catheter to expand the wall.^17^ Figure 8(c) shows a cross-section of the malleus handle (H) attached to several strands of connective tissue (L)—likely ligaments that appear to connect it to both the tympanic membrane (TM) and the attic wall of the middle ear.

Although we can clearly discern many morphological features within the middle ear, shadowing from structures such as the ossicles limits us from imaging the whole middle ear. Incorporating some steering mechanism into the distal end of the catheter may alleviate this issue. Volume images measured from multiple orientations could then be fused to create a comprehensive view of the middle ear. Similarly, we could also fuse volume images acquired through the ear canal to get a more complete volume including all of the tympanic membrane. Moving forward, imaging human cadavers in a similar manner will be essential to evaluate how shadowing affects OCT volumetric images and to identify additional engineering requirements for achieving a viable solution for comprehensive middle ear imaging.

**Fig. 9:**
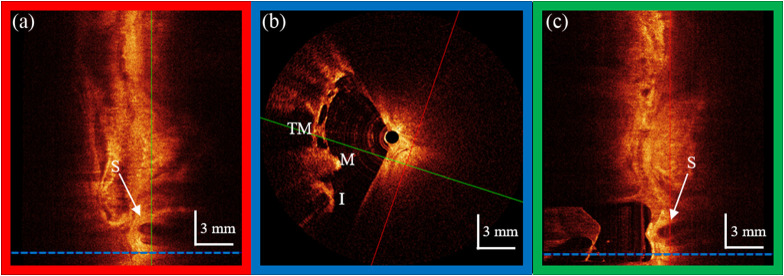
Cross-sectional OCT images of the cochlea scala (S) in the volumetric images collected with the catheter endoscope. Panels (a) and (c) are longitudinal (pullback) cross-sections that show the scala within the cochlea and the subsurface layers of the Eustachian tube. Panel (b) shows the transverse (rotational) cross section showing the incudomalleolar joint where the incus (I) and malleus (M) meet with the tympanic membrane (TM) above the ossicles. These frames are related cross-sectional images where the color of the frame around each image matches the color of the scout lines in the other two frames. These scout lines show where the frame’s cross-sectional image comes from.

Given the catheter endoscope’s close proximity to the cochlear promontory, our images were examined for cross-sectional views of the cochlea. Due to the thickness of the surrounding bone, there was no expectation to visualize the interior of the cochlea. However, we were surprised to find clear cross-sectional images revealing the scala within the cochlea, as illustrated in Fig. 9. Figure 9(a) shows a cross-sectional image of the scala of the cochlea and the layers of tissue surrounding the Eustachian tube. Figure 9(b) shows a cross-section of the ossicles and the tympanic membrane for anatomical orientation, and Fig. 9(c) shows the clearest cross-sectional image of the scala of the cochlea. This is important because there is currently no way to image the inner ear in a human. If this can be repeated in a human cadaver, it may prove to be a viable approach for *in vivo* imaging of the human inner ear.

Although the catheter endoscope performed well in the porcine model, additional tuning may be necessary for future human cadaver imaging experiments. From the porcine study, we learned that the catheter’s stiffness must be optimized to balance flexibility and pushability: it needs to be flexible enough to navigate the sharp turn near the isthmus of the Eustachian tube, yet stiff enough to traverse the narrow isthmus and reach the middle ear. Excessive flexibility made it nearly impossible to advance the catheter through the isthmus, whereas excessive stiffness hindered navigation through the turn and precise positioning within the middle ear. The human Eustachian tube is slightly narrower than that of the porcine model, potentially presenting additional challenges. In this study, we selected the FEP catheter tubing wall thickness to achieve the stiffness that allowed for successful imaging, but adjustments to the wall thickness may be needed to accommodate anatomical differences in future experiments.

We were pleased to observe that six out of the seven fabricated catheter endoscopes performed as expected, with only minor deviations in performance. As shown in Table 2, the results demonstrate that 3D-printing these optical probes yielded highly consistent structures across the batch. The mean radii of curvature for the set of 10 reflective lenses precisely matched the design specifications from OpticStudio, and the standard deviations for each radius were well below 1% of the mean—indicating excellent manufacturing consistency. This outcome underscores the strength of the iterative optimization process employed in developing these probes. Had the performance fallen outside specification, the design could have been readily adjusted and reprinted, enabling rapid iteration to achieve the desired results.

Another notable outcome was the close agreement between the measured spot sizes and the simulated values, reaffirming the effectiveness of combining optics simulation software with 3D printing for achieving high-precision custom optics. The single outlier among the set of probes was likely the result of human error during the catheter endoscope assembly. This conclusion is supported by the high consistency in printed lens geometry shown in Table 2. Although the exact cause of the failure remains unclear, it is suspected that a slight misalignment between the fiber and the reflective lens or an undetected defect in the catheter tubing may have contributed to the deviation.

Looking back at the fabrication of these catheter endoscopes, we see a promising future using the 2PP technique to fabricate the micro-optics for catheter endoscopes. The ability to tune the design of the reflective lens and take an iterative approach to produce many high-performance probes in a high-precision fashion is just a glimpse into the future marriage of optics and additive manufacturing. If anything needed to be adjusted, it only required a reoptimization in OpticStudio to produce the new STL file before printing the new optic. These types of adjustments are typically very time consuming or costly in traditional micro-optics fabrication, especially when the optics are complex. Additive manufacturing also provides the opportunity to design complex optics, which are difficult or impossible to produce using standard manufacturing techniques, which could lead to further innovation in the future. The 2PP technique has been advancing rapidly, achieving significant enhancements in both print speed and quality, with further improvements anticipated soon.

## 4 Conclusion

We report the successful development of a reflective lens catheter endoscope, created using the 2PP additive manufacturing approach to design and fabricate the required miniature optics. Of the seven reflective lenses tested, all but one of them met all performance specifications, with the entire set taking only 4 h to print and coat. The catheter endoscope achieved its intended purpose by successfully imaging the middle ear with high resolution through the Eustachian tube. Unexpectedly, we discovered that this approach also enables imaging of the cochlea. The images revealed sections of two turns in the porcine cochlea, suggesting the potential for cochlear imaging in human patients—a capability currently unavailable.

## Data Availability

OCT volume images are available upon reasonable request to the corresponding author. No specialized code was used for this research.
